# Social marketing of low dead space syringes in Vietnam: findings from a 1-year pilot program in Hanoi, Thai Nguyen, and Ho Chi Minh City

**DOI:** 10.1186/s12954-015-0049-y

**Published:** 2015-05-30

**Authors:** Ngo Thi Thanh Huong, Gary Mundy, Josselyn Neukom, William Zule, Nguyen Minh Tuan, Nguyen Minh Tam

**Affiliations:** Population Services International, Washington, USA; Research Triangle Institute International, Durham, USA; Vietnam Administration for HIV/AIDS Control/Ministry of Health, Hanoi, Vietnam

**Keywords:** Low dead space syringes, Injection drug use, Social marketing, HIV prevention, Vietnam

## Abstract

**Background:**

Although a growing body of evidence suggests that low dead space syringes may reduce the risk of human immunodeficiency virus (HIV) and Hepatitis C virus infection associated with sharing syringes among people who inject drugs, there is little evidence of effective approaches to motivate people who inject drugs (PWID) to shift from high to low dead space syringes.

**Methods:**

Using a mix of consumer and trade marketing approaches, informed by rapid assessments of both the syringe market and PWID preferences, practices, and behaviors in Hanoi and Ho Chi Minh City, Population Services International (PSI) Vietnam piloted an intervention to increase the use of low dead space syringes (LDSS) in the three provinces of Hanoi, Ho Chi Minh City, and Thai Nguyen, where an estimated 31 % of PWID are HIV positive and 58 % are living with hepatitis C virus (HCV).

**Results:**

This paper provides a summary of the social marketing activities implemented and results achieved by PSI Vietnam during an initial 1-year pilot period from December 2012 to December 2013 in these three provinces to explore their effectiveness in motivating PWID to use low dead space syringes.

We found major increases in sales of LDSS accompanied by increases in reported use and consistent use of LDSS among PWID in the three provinces included in the pilot program and a positive and independent association (odds ratio (OR) 21.08; 95 % confidence interval (CI) 10.6–27.3) between LDSS use and exposure to social marketing activities. We also found that LDSS use had a stronger association with perceptions of LDSS product quality than with perceptions regarding LDSS potential to reduce HIV transmission risk and use.

**Conclusions:**

We conclude that social marketing interventions have an important role to play in widening access to and the use of LDSS for PWID, as they address the need for PWID to find LDSS when and where they need them and also promote the benefits of LDSS use to PWID. High coverage of these activities among PWID appears to be the key in achieving these successes.

## Background

A growing body of evidence suggests that low dead space syringes (LDSS) may reduce risk of HIV and hepatitis C virus (HCV) infection associated with sharing syringes among people who inject drugs (PWID) [1–6]. In laboratory experiments that simulated the process of injection and rinsing with water, high dead space (i.e., standard needle and syringe combinations) syringes retained over 1000 times more blood than that retained by low dead space syringes. Although very few studies have reported data on the types of needles and syringes shared, those that have been done have found an association between a history of sharing high dead space syringes and testing positive for HIV and HCV but no association between a history of sharing low dead space syringes and testing positive for HIV or HCV.

Ecological data collected from needle and syringe programs (NSP) in Europe and Asia suggest that HIV prevalence is relatively low in cities where only low dead space syringes are used, while in contrast, HIV prevalence was varied greatly in cities where substantial proportions of PWID used high dead space syringes [6]. Simulation models of injection-related HIV epidemics predicted that risk of an epidemic among PWIDs would be curtailed if only LDSS are used. In other models, changing PWID from high dead space to low dead space syringes reverses HIV epidemics. There is also some evidence that LDSS may offer protection against HCV infection. In models that adjusted for a large number of potential confounders, a history of sharing high space syringes was associated with significantly increased odds of testing positive for HCV. In contrast, the same model found no association between a history of sharing syringes and testing positive for HCV among people who had never used a high dead space syringe [4].

In a systematic review of published evidence of the potential efficacy of LDSS in reducing transmission risk, Vickerman and colleagues concluded that there was a ten-fold average difference in risk of HIV transmission, lower than some of the earlier modeled estimates, but still a substantial reduction in risk compared with high dead space syringes (HDSS) and with the potential to positively impact HIV prevalence rates [3].

Based on a combination of biological plausibility and data from a number of studies, the World Health Organization included a recommendation for needle syringe programs to offer LDSS in its guidance on viral hepatitis B and C prevention among PWID that was issued in 2012 [2].

There are still many questions remaining about the implementation of such an LDSS program component, such as the uncertainty of PWID’s willingness to switch from high dead space needles and syringes to low dead space needles and syringes—particularly because LDSS are not currently manufactured in all needle and syringe (N/S) product sizes and specifications currently used by PWID. User preferences around barrel size and detachable needles have been identified as powerful factors shaping syringe choice made by PWID [6]. Drug preparation practices (e.g., removing the needle to draw drug solution out of a spoon or cooker) and practices for dividing liquefied drug solution (e.g., frontloading or backloading) influence preferences for detachable needles. Location of the injecting site on the body influences preferences regarding the length and gage (i.e., diameter) of needles [4, 7, 8].

There is a need to evaluate the real-life effectiveness of strategies that promote the use of LDS syringes among populations that are currently using HDS syringe types, taking into account an understanding of current use and preference for syringe types among PWID [4, 9].

### Description of PSI Vietnam’s social marketing pilot program to improve access and motivate use of LDSS among PWID in select provinces

Population Services International (PSI) Vietnam conducted a N/S market assessment in Hanoi and Ho Chi Minh City (HCMC) during 2011 [10] to assess the availability, pricing, and late-night accessibility of LDS syringes in these two locations. The market assessment consisted of an audit of multiple points along the N/S supply chain—starting with shooting galleries and ending with N/S manufacturers.

The 2011 market assessment, along with a survey conducted in 2012 [11], found substantial differences in the availability and use of LDSS in these locations. In Ho Chi Minh City (HCMC), LDS syringes were stocked at pharmacies and interviews with PWID combined with observations of discarded N/S at shooting galleries revealed high degrees of awareness and use of LDS syringes among PWID in HCMC, with almost all being frequent users. The B Braun Omnican^©^ 1-ml LDSS product also known as “Kim Dau Do” was found to be the most commonly used LDS syringe product among PWID in HCMC. Access gaps were identified, however, including late-night access when pharmacies close, as well as day-time barriers in cases where pharmacy operators refused to sell to PWID.

By contrast, the assessment found no availability of LDS syringes in Hanoi, indicating more significant market access gaps to LDS products among PWID in the north. At the time of the 2011 market assessment, almost no PWID in Hanoi had heard of LDS syringes, knew where to buy them, or had any experience using. None of the pharmacies surveyed stocked the B Braun Omnican^©^ 1-ml LDS syringe product popular among PWID in HCM.

While pharmacies are the most common source of syringes, many PWID felt that they were not welcome customers at these outlets. Mystery shopper exercises confirmed feedback from PWID that pharmacy operators limit clean N/S access among PWID by refusing to sell a single N/S, explaining ‘we only sell to doctors who buy full dispensers.’ Given the risk of arrest, PWID prefer opportunities to discreetly purchase 1 N/S at a time.

Qualitative research conducted by PSI Vietnam in 2012 revealed that syringe size as well as overall quality are the key factors influencing choice of syringe. This qualitative research indicated that PWID in the north may be hesitant to switch from a 3–5-ml HDS syringe with a 1-in.-long needle to a 1–2-ml LDS syringe with a 1-in.-long needle, given drug mixing and sharing practices among northern PWID. Interviews with PWID in Hanoi revealed a practice of preparing, measuring, and dividing drug solutions between two or more people. This practice, known as “syringe-mediated drug-sharing,” is common in many countries [12]. Another reason for preferring the 3–5-ml syringes is that some poor-quality heroin may require larger volumes of water to dissolve it.

In HCMC, we found that the preference for LDS syringes was driven by perceptions of the quality of the product—the needle produced less scarring, caused less pain, and was easy to use—and the fact that the needle is easily detached from the barrel. This latter feature made it easier for PWID to access substances that may become stuck in the barrel and was perceived to reduce the risk of drug loss.

Using the insights from this formative work, PSI Vietnam developed and tested a social marketing pilot program. Pilot activities differed between HCM and Hanoi in line with variation in PWID behavior and preferences and related, regional market context differences. In the northern provinces (Hanoi and Thai Nguyen), PSI distributed LDS syringes through both pharmacies as well as non-pharmacy outlets accessible to PWID, given the absence of any LDS syringe availability among pharmacies or other commercial outlets in the north pre-pilot. However, in HCM, where commercial access to LDS syringes was established at select pharmacies pre-pilot, the project focused on improving late-night access to LDS syringes by establishing non-pharmacy outlets and motivating pharmacy operators to sell to PWID.

The type of non-pharmacy outlets utilized in the pilot program also varied by region based on pre-established patterns of behavior among PWID. For example, tea stalls were the primary non-pharmacy outlet utilized in Hanoi and Thai Nguyen given the common practice of drinking tea in the north. In HCM, coffee shops and cigarette sellers were utilized. Images of convenient LDSS outlets—featured in behavior change communication (BCC) materials—varied as a result. Language used to communicate regarding the benefits of LDS syringes also varied slightly, in line with regional differences delineating northern and southern dialects of Vietnamese.

The pilot program was designed to remove barriers to LDS syringes by developing an alternative distribution channel through tea stalls and other outlets open late at night when pharmacies are closed and when free N/S distribution channels are not operational. In addition, the pilot program used print materials, community events, and face-to-face communication tools to promote the benefits of the 1-ml LDS syringe product in terms that resonated with PWID, emphasizing less pain and scaring due to high quality needle and less risk of drug loss due to low dead space. Messages promoting the benefits of LDS syringes were disseminated through outlets stocking LDS syringes, events organized by PSI, and through outreach networks managed by the Provincial AIDS Committees. Outdoor billboards promoting non-sharing of any N/S were placed near shooting galleries.

To improve access to LDS syringes, PSI Vietnam connected LDS syringe supplier BBVN (B Braun Vietnam Co., Ltd.) with its targeted distribution network covering tea stalls, coffee shops, truck stations, motorcycle taxi drivers (xe om), pharmacies near shooting galleries, and other outlets accessible to PWID. BBVN provided significant contributions to the partnership including a price reduction, donated stock for distribution to PWID unable to pay the commercial price, and cost-share contributions to trade marketing materials.

With a small, dedicated LDS syringe sales team (one agent per province), PSI Vietnam identified commercial outlets accessible to PWID and motivated these outlets to stock, display, and promote LDSS. This effort to improve convenient access to LDS syringes through non-pharmacy outlets was a major focus of the pilot program in all three provinces, whereas distribution coverage also included pharmacies in Hanoi and Thai Nguyen, given the limited commercial distribution reach pre-pilot. By December 2013, PSI was able to motivate 874 commercial outlets in 14 districts across the three pilot provinces to stock LDSS. Focus districts were identified in consultation with the Provincial AIDS Committees and the Departments of Health in each province.

Targeted distribution and promotion of LDS syringe was initiated first in HCMC in September 2012, expanded to Hanoi in December 2012, and launched in Thai Nguyen in March 2013.

Trade marketing efforts were utilized to motivate outlets to stock and display LDSS, as well as to collect used N/S. Battery-operated lights were distributed to these outlets and were used to 1) reward these outlets for stocking LDS syringes, 2) increase overall business viability as more light at night leads to more sales/revenue across all products, and 3) send a subtle signal to PWID that LDS syringes were available for sale at those outlets.

The Nevershare—a larger 2-ml syringe—was integrated into PSI’s targeted LDS syringe distribution system in August 2013, and BCC materials were designed to promote the Omnican and the Nevershare products as two LDS syringe options for PWID. However, uptake of the Nevershare product was limited in comparison to the Omnican product due to the higher price of the Nevershare syringe and perceptions that the Omnican product was a higher quality product.

To introduce the LDS syringe product to PWID in the north who were previously unaware of this product and its benefits, PSI Vietnam used a variety of targeted communication channels to emphasize the benefits of the 1-ml Omnican and 2-ml Nevershare LDS syringe products, including their lowered risk of losing and wasting drugs as a result of less dead space, decreased incidence of pain or scaring due to high quality needle, and reduced risk of both HIV and HCV transmission.

Key messages were disseminated using posters and leaflets, community events at venues accessible to PWID, and face-to-face communication through PWID peer and outreach networks linked to Provincial AIDS Committees and PWID-focused civil social organizations (CSOs). These LDS syringe behavior change communication activities were implemented in 14 districts across the three provinces in 2013.

## Methods

We used data from two cross-sectional surveys of males who reported having injected in the past 3 months. The first round of the survey was conducted in December 2012 in Hanoi and Ho Chi Minh City. The second round of the survey was conducted in December 2013 in Hanoi, Ho Chi Minh City, and Thai Nguyen province. In both rounds of the survey, and in all locations, participants were recruited through respondent-driven sampling (RDS).

RDS is a chain-referral method commonly used to recruit hard-to-reach populations, including people who inject drugs [13]. RDS relies on the assumption that, given sufficiently long referral chain, the sample composition becomes stable or reaches “equilibrium,” which results in a sample that has the characteristics of a probability sample [13].

We started by recruiting four seeds in each study city. We diversified seeds based on length of time injecting, exposure to harm reduction programs, and education. We gave each seed three coupons and asked him to recruit three peers from his social network to participate in the study. The recruitment coupons were uniquely numbered, which allowed us to link seeds to their referrals and track the length of recruitment chains. Coupon numbers were also linked to the questionnaire and were used as a code for analysis. Respondents received 50,000 VND (approximately US$2.50) for each person they successfully recruited that met the screening criteria.

Respondent numbers for each site over the two rounds of the study were as follows: Hanoi *n* = 300 (2012) and *n* = 400 (2013), Ho Chi Minh City *n* = 300 (2012) and *n* = 400 (2013), and Thai Nguyen *n* = 280 (2013). We collected data in Thai Nguyen in 2013 only. When the survey was conducted in 2012, Thai Nguyen was not part of the social marketing program. We collected data through face-to-face interviews in private locations where respondents’ privacy could be protected.

The study was designed to test the hypotheses that a social marketing pilot program would result in the following:

1. An increase in the awareness of LDS syringes among PWID,
2. An increase in the use of LDS syringes among PWID, and
3. That there would be a significant, independent, and positive association between exposure to LDS syringe demand creation activities implemented through the social marketing pilot program and use of LDS syringes.

A sample size of 1080 PWID—out of an estimated total 58,512 PWID living in the three provinces—was estimated as being necessary to test these hypotheses. The sample distribution across provinces was selected based on consideration of the following: the need to ensure the integrity of provincial-level RDS samples, ensuring that they are of sufficient size to allow for a minimum of four waves of recruitment from each seed, estimates of the size and diversity of the PWID population in each sample province, precision of estimates, and the resources and time available for the study.

We also used PSI Vietnam’s LDS syringe sales data between September 2012 to December 2013 to inform analysis of changes in access to and the use of LDS syringes during the pilot program. PSI’s MIS data is managed through an online database, updated daily on the basis of sales reports submitted by field-based sales agents, and checked weekly by the Hanoi-based monitoring team, with a monthly cross-check against financial reports related to sales during the same time period.

### Study population

Inclusion criteria for study participation were as follows: respondent has injected in the last 1 month and has been injecting for at least 3 months but not longer than 10 years, respondent currently lives in the city/province sampled, and respondent is not currently employed as a peer educator or outreach worker. Potential respondents were excluded from the study if they were incapacitated due to drug or alcohol use and not able to complete an interview within 2 hours of being sampled. All potential respondents meeting the screening criteria were then asked to provide informed consent to participate. A structured questionnaire was used to collect data and administered to respondents by interviewers.

Respondents were interviewed in private rooms rented specifically for the study. Interview venues were selected based on proximity to PWID ‘hot spot’ areas in each province to minimize travel time and costs for respondents.

### IRB review

The Hanoi School of Public Health Institutional Review Board (IRB) reviewed and authorized this study prior to data collection: IRB registration No: 168/2013/YTCC-HD3 in response to IRB application No: 013-168/DD-YTCC.

### Measures for logistic regression model

We used injecting with an LDS syringe in the past month as the dependent variable for logistic regression. We tested association with the independent variable, exposure to demand creation activities operated under the social marketing pilot program. This was defined as having been exposed to messaging promoting LDS syringes through print or other channels used by the pilot program.

### Statistical analyses

Two-proportion *z* tests were used to identify significant differences in demographic profiles and behaviors related to injecting and syringe use between 2012 and 2013 in Hanoi and Ho Chi Minh City and between behaviors observed in each study location.

To obtain effect estimates for exposures to social marketing program accounting for potential confounders, we constructed a multivariable logistic regression model, using data from Hanoi and Thai Nguyen, where there were a sufficient number of cases that were not using LDS syringes. Data from HCMC were not included in the multivariate analysis as there were an insufficient number of respondents that did not report use of LDS syringes.

We used a *p* value of <0.05 in the bivariate analyses to determine whether a secondary explanatory variable was considered a potential confounder in the relationship between exposure to demand creation activities promoting LDS syringes and use of LDS syringes in the past 1 month. We ran a correlation matrix to check for multicollinearity between variables, using a correlation coefficient value of >0.70 as the cut point. Where collinearity was found, we removed the variable with the weakest correlation with LDS syringe use in the past 1 month from the full multivariable model.

The full model was then constructed through first running the model with all significant variables from the bivariate analysis. We then reduced the model down so that only significant variables (*p* < 0.05) were included, and we then re-entered dropped variables one at a time. If each variable improved the model through omnibus chi square statistic and individual *p* values, then it was retained in the model. Variables that did not improve the model were dropped.

## Results

**Fig 1:**
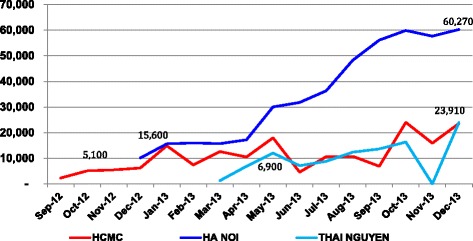
Monthly sales of LDSS syringes in Ho Chi Minh City, Hanoi, and Thai Nguyen

We observed monthly sales of LDS syringes increased across all three provinces (Fig. 1). The average monthly LDS syringe sales to outlets accessible to PWID in Hanoi increased from 10,000 units in December 2012 to around 60,000 in December 2013. Monthly sales in HCMC increased from 5100 in September 2012 to around 23,000 in December 2013, and sales in Thai Nguyen increased from 6900 in April 2013 to a little over 23,000 in December 2013.

Injecting behaviors observed in the December 2012 and December 2013 samples from Hanoi and HCMC and the 2013 sample from Thai Nguyen are shown in Table 1. We saw a major increase in awareness of LDS syringes in Hanoi (16.5 % in 2012; 43.2 % in 2013) and a significant increase in use of LDS syringes (0.3 to 5.8 % reporting having ever used and 0.1 to 4.3 % reporting having used in the past 1 month). We saw a small but significant increase in respondents from Hanoi reporting consistent use of LDS syringes—used at every injection in the past 1 month—from 0.1 % in 2012 to 2.3 % in 2013.

**Table 1:** Injecting behaviors among male injecting drugs by provinces, 2012 and 2013

Indicators	Hanoi			HCMC			Thai Nguyen	*p* value between cities in 2013 samples
	2012 *n* = 300	2013 *n* = 400	*p* value	2012 *n* = 300	2013 *n* = 400	*p* value	2013 *n* = 280	
Awareness of LDSS (ever seen or heard about LDSS)	16.5	43.2	0.000	96.8	99	.008	60.7	0.000
Awareness about LDSS benefits:								
Aware that using LDSS may reduce the risks of HIV and HCV infection	NA	12.0		NA	31.4		62.2	0.000
Aware that LDSS can minimize risk of drug lose		14.6			94.4		56.6	0.000
Aware that LDSS can reduce pain		12.0			96.4		57.5	0.000
Aware that LDSS can reduce scarring		11.7			95.7		58.0	0.000
Use of LDSS:								
Ever used LDSS	0.3	5.8	0.000	100	100	1	57.9	0.000
Used LDSS in the last 1 month	0.1	4.3	0.000	93.0	98.7	0.001	33.8	0.000
Consistently use in the last 1 month	0.1	2.3	0.021	88.0	93.2	0.023	8.1	0.000
Reasons for not using LDSS in the last 1 month among those who did not use LDSS	*n* = 299	*n* = 384		*n* = 28	*n* = 6		*n* = 113	
Unaware of LDSS	83.5	45.6	0.000	0	0		34.4	0.000
Unaware of outlets selling LDSS	16.3	4.1	0.000	0	0		64.1	0.000
No outlet selling LDSS close to injecting sites	6.8	2.0	0.001	58.3	33.3	0.265	11.2	0.000
LDSS is too expensive	1.2	0	0.031	16.7	16.6		0.7	0.000
LDSS size is not big enough	4.9	3.6	0.399	0	0	1	11.4	0.000
It is difficult to share drug with LDSS	2.8	2.7	1	0	0		5.1	0.015
It is difficult to find LDSS at night	1.7	0	0.010	31.4	16.6	0.459	0	
Needle-sharing behavior								
Sharing needle in the last 12 months	11.3	3.5	0.000	19.1	12.9	0.030	7.8	0.000
Sharing needle in the last 1 month	5.1	2.3	0.028	14.8	8.2	0.003	5.5	0.001

In the Hanoi sample, we saw a significant decline between 2012 and 2013 in those reporting that their reason for not using an LDS syringe was because they were not aware of LDS syringes (83.5 % in 2012 to 45.6 % in 2013). We saw a significant decline in those reporting their reason for not using was because they did not know the location of outlets selling LDS syringes (16.3 % in 2012 to 4.1 % in 2013) or that there were no outlets selling LDS syringes close to the place(s) where they inject (6.8 % in 2012 to 2 % in 2013).

Among the HCMC sample, 100 % of those sampled in both rounds reported having previously injected with an LDS syringe. We saw a significant increase in those reporting having used an LDS syringe in the past 1 month (93 % in 2012 to 98.7 % in 2013) and a small but significant increase in those reporting consistent use of LDS syringes—used at every injection in the past 1 month—from 88 % in 2012 to 93.7 % in 2013. We did not find a sufficient number of people that were not using LDS syringes to allow us to test for differences between 2012 and 2013 in the reasons given for not using.

In Thai Nguyen sample (2013), a little over 60 % reported awareness of LDS syringes, 57.9 % reported ever using LDS syringes, and 33.8 % reported using in the past 1 month. Respondents (8.1 %) reported using LDS syringes at every injection in the past 1 month. The most commonly cited reasons for not using LDS syringes were unawareness of outlets selling LDS syringes (64.1 %) and being unaware of LDS syringes (34.4 %).

We found a large variation in the proportion of respondents reporting awareness that their use of LDS syringes may reduce the risks of HIV and HCV infection (12 % in Hanoi, 31.4 % in HCMC, and 62.2 % in Thai Nguyen). We found that awareness of wider product benefits of LDS syringes was more prevalent in Ho Chi Minh City and Thai Nguyen than in Hanoi. Awareness that LDS syringes can minimize drug loss was 14.6 % in Hanoi, 94.4 % in HCMC, and 56.6 % in Thai Nguyen. Awareness that LDS syringes can reduce pain was 12 % in Hanoi, 96.4 % in HCMC, and 57.5 % in Thai Nguyen. Awareness that LDS syringes can reduce scarring was 11.7 % in Hanoi, 95.7 % in HCMC, and 58 % in Thai Nguyen.

We found significant reductions in reported needle/syringe sharing between the 2012 and 2013 surveys in Hanoi and HCMC. In Hanoi, we found a reduction in reported sharing in the past 12 months from 11.3 % in 2012 to 5.3 % in 2013 and a reduction in reported sharing in the past 1 month from 5.1 % to 2.3 %. In HCMC, we found a reduction in reported sharing in the past 12 months from 19.1 % (2012) to 12.9 % (2013) and a reduction of reported sharing in the past 1 month from 14.8 % (2012) to 8.2 % (2013). In Thai Nguyen, we found that 7.8 % reported sharing a needle/syringe in the past 12 months and 5.5 % reported sharing in the past 1 month.

The drug use profile of 2012 and 2013 samples from Hanoi and HCMC are shown in Table 2, together with the drug use profile of those sampled in Thai Nguyen in 2013. There were no significant differences between the 2012 and 2013 samples (for Hanoi and HCMC) in the average length of injecting (4.6 years in 2012 vs. 4.7 years in 2013); the average number of injection times per day (2.1 in 2012 vs. 2.3 in 2013 in Hanoi and 2.3 in 2012 vs. 2.4 in 2013 in HCMC); the types of drugs used; or the percentage paying for needles/syringes, rather than getting them for free (85.4 % reported paying for N/S in 2012 vs. 84.3 % in 2013). However, during the same time period, there was a significant increase in the proportion reporting purchasing from non-pharmaceutical commercial outlets including tea stalls, cigarette sellers, and motorcycle taxi drivers (1.9 % in 2012 vs. 7.4 % in 2013).

**Table 2:** Needle/syringe sources in Hanoi, HCMC, and Thai Nguyen—2012 and 2013

Injecting indicators	Hanoi			HCMC			Thai Nguyen	
	2012 *n* = 300	2013 *n* = 400	*p* value	2012 *n* = 300	2013 *n* = 400	*p* value	2013 *n* = 280	
Average length of injecting (year)	5.1	4.7	1	4.3	4.5	1	5.3	1
Average number of injections each day (time)	2.1	2.3	1	2.3	2.4	1	1.8	1
Payment for needle/syringe used in the last 1 month (%)								
Purchased all needle/syringes used	73.4	85.3	0.000	77.1	86.1	0.000	79.1	0.042
Some purchased, some free	22.1	12.1	0.000	17.2	11.7	0.038	15.7	0.122
Replied on free needle/syringes used	4.5	2.6	0.170	5.7	2.2	0.015	5.2	0.030
Needle/syringe supplies among those purchased N/S in the last 1 month (%)	*n* = 285	*n* = 364		*n* = 282	*n* = 380		*n* = 263	
Pharmacy	90.5	88.1	0.421	89.6	87.5	0.436	93.8	0.012
Injecting mate	8.6	12.5	0.095	11.1	10.8	0.677	2.1	0.000
Tea stall/cigarette seller/motorbike driver	1.9	6.4	0.003	1.3	6.9	0.001	5.3	1
Sources of needle/syringe late at night in the last 1 month (%)								
Pharmacy in hospital	NA	11.4		NA	2.8		2.1	0.000
Injecting mate		34.5			7.3		4.1	0.000
Tea stall/cigarette seller/motorbike driver		33.7			8.5		21.5	0.000
Re-used N/S		8.9			67.9		62.5	0.000

We found that late at night, after pharmacies have closed, PWID reported re-using syringes already on hand. The following most common source of N/S late at night were tea stalls, cigarette sellers, and motorcycle taxi drivers—outlets which prior to the PSI pilot program were not providing LDS syringes. We found that re-used syringes were more common among the Thai Nguyen sample than in the samples from Hanoi and HCMC.

Table 3 shows the level of exposure to demand creation activities promoting LDS syringes found in the 2013 samples from Hanoi, HCMC, and Thai Nguyen. We found higher reach of intervention programs providing information on LDS syringes and HIV prevention messages in Thai Nguyen (30.2 %) compared with Hanoi (10.7 %) and HCMC (11 %). Similarly, we found higher exposure to leaflets/posters and social marketing events promoting LDS syringes in Thai Nguyen (24 and 11 %) compared with HCMC (7.1 and 1.6 %) and Hanoi (7.6 and 3.6 %). We found higher exposure across all three sites to messages around needle sharing (as compared with messages about LDS syringes), with higher exposure in Thai Nguyen (76.1 %) compared with HCMC (65.3 %) and Hanoi (63.2 %).

**Table 3:** Social marketing activity exposure by provinces in 2013

Social marketing activities	Hanoi *n* = 400	HCMC *n* = 400	Thai Nguyen *n* = 280	Total *N* = 1080
Exposure to any messages on LDSS	16.7	10.1	56.4	18.3
Exposure to messages on LDSS by channels				
LDSS inter-personal communication tools used by outreach workers	10.7	11.0	30.2	13.1
Leaflet/poster	7.6	7.1	24.0	9.2
Social marketing events	3.6	1.6	11.0	3.1

The results of bivariate analyses are presented in Table 4. Using LDS syringes in the past 1 month was significantly and positively associated with injecting frequency (odds ratio (OR) 1.731; 95 % confidence interval (CI) 1.101–2.665), being stopped by the police in relation to drug use (OR 1.65; 95 % CI 1.116–2.452), belief that LDS syringes are easily hidden form the police and other non-drug users (OR 1.718; 95 % CI 1.290–2.015), awareness that LDS syringes can be accessed late at night (OR 2.733; 95 % CI 1.052–3.219), LDS syringe use is encouraged by their drug-using friends (OR 2.341; 95 % CI 1.374–2.816), reporting that LDS syringes are affordable (OR 1.732; 95 % CI 1.034–2.310), awareness of non-health benefits of LDS syringe use (OR 1.732; 95 % CI 1.034–2.310), awareness that LDS syringes can reduce the risk of HIV/HCV infection (OR 1.446; 95 % CI 1.263–1.655), and exposure to social marketing activities promoting use of LDS syringes (OR 23.08; 95 % CI 13.26–40.16).

**Table 4:** Bivariate analysis of factors associated with LDS syringe use in the last month among surveyed IDUs in the North (*n* = 680)

Characteristics	Total *n* (%)	Used LDSS in the last month *n* (%)	Odds ratio (95 % CI)	*r*	*p* value
Age	680	143	1.148 (0.357–3.767)	0.046	0.254
Education level					
Never been at school/primary school (ref)	32	3 (2.1 %)	0.204 (0.019–2.216)		
Secondary school	311	60 (41.9 %)	0.737 (0.398–1.365)	0.217	0.192
High school or above	337	73 (51.0 %)		0.314	0.332
Income	680	143	0.905 (0.757–1.082)	0.097	0.273
Injection frequency in the past week	680	143	1.731 (1.101–2.665)	0.538	0.017
Ever been stopped by police relating drug use					
Yes	251	58 (40.5 %)	1.655 (1.116–2.452)	0.750	0.006
No	429	85 (59.5 %)			
Sharing N/S in the last 12 months					
Yes	35	8 (5.6 %)	1.869 (.663–5.271)	0.049	0.230
No	645	135 (94.4 %)			
Self-reported HIV status					
Positive	81	6 (4.2 %)	1.397 (0.542–3.297)	0.031	0.444
Negative or unknown	599	137 (95.8 %)			
Believed that LDSS is easily hidden from police and other non-drug users					
Yes	186	56 (24.4 %)	1.718 (1.290–2.015)	0.654	0.027
No	422	87 (61.6 %)			
Aware that LDSS is easily accessed at late night					
Yes	146	39 (27.3 %)	2.733 (1.052–3.219)	0.317	0.016
No	534	104 (72.7 %)			
LDSS is encouraged to use by injecting mates					
Yes	136	63 (44.0 %)	2.341 (1.374–2.816)	0.421	0.013
No	544	80 (56.0 %)			
LDSS is affordable					
Yes	98	16 (11.2 %)	1.732 (1.034–2.310)	0.360	0.027
No	582	127 (88.8 %)			
Awareness of LDSS non-health benefits					
Yes	238	95 (66.4 %)	4.231 (2.734–4.710)	0.713	0.001
No	442	48 (33.6 %)			
Aware that LDSS can minimize the risk of HIV and HCV infection					
Yes	176	54 (37.7 %)	1.446 (1.263–1.655)	0.523	0.012
No	514	89 (62.3 %)			
Exposure to LDSS social marketing activities					
Yes	145	111 (77.6 %)	23.08 (13.26–40.16)	0.789	0.001
No	535	32 (22.3 %)			

*CI* confidence interval, *r* Pearson’s correlation coefficient

The results of multivariable analysis are shown in Table 5. Use of low dead space syringes in the past 1 month were significantly and independently associated with exposure to social marketing activities promoting use of LDS syringes (OR 21.08; 95 % CI 10.60–27.3), belief that LDS syringes are easily hidden form the police and other non-drug users (OR 1.32; 95 % CI 1.09–1.91), awareness that LDS syringes can be accessed late at night (OR 2.03; 95 % CI 1.05–2.73), LDS syringe use is encouraged by their drug-using friends (OR 1.80; 95 % CI 1.32–2.16), reporting that LDS syringes are affordable (OR 1.22; 95 % CI 1.03–1.73), awareness of non-health benefits of LDS syringe use (OR 3.19; 95 % CI 1.05–3.31), and awareness that LDS syringes can reduce the risk of HIV/HCV infection (OR 1.30; 95 % CI 1.16–1.65).

**Table 5:** Multivariable regression analysis of factors associated with LDSS use in the last month among PWID in Hanoi and Thai Nguyen (*n* = 680)

Characteristics	Odds ratio	(95 % CI)	*p* value
Believed that LDSS is easily hidden from police and other non-drug users (yes vs. no)	1.32	(1.09–1.91)	0.015
Aware that LDSS is easily accessed at late night (yes vs. no)	2.03	(1.05–2.73)	0.007
LDSS is encouraged to use by injecting mates (yes vs. no)	1.80	(1.32–2.16)	0.013
LDSS is affordable (yes vs. no)	1.22	(1.03–1.73)	0.027
Awareness of LDSS non-health benefits (yes vs. no)	3.19	(1.05–3.31)	0.001
Aware that LDSS can minimize the risk of HIV and HCV infection (yes vs. no)	1.30	(1.16–1.65)	0.022
Exposure to LDSS social marketing activities (yes vs. no)	21.08	(10.6–27.3)	0.001

Adjusted by age, education, income, and injection frequency in the past week

## Discussion

We found that, after a staggered implementation of a pilot program to improve convenient access to and promote informed demand for LDS syringes across three provinces during a 1-year period, there was a 4–6-fold increase in both sales of LDSS and reported use of LDS syringes among PWID in the pilot provinces. We found increased sales and increased reported use of LDS syringes, particularly in Hanoi and Thai Nguyen provinces, where there had been no regular supply of LDS syringes to pharmacies prior to the pilot program and near non-existent reported use of LDS syringes among injectors in those areas.

Importantly, we found a very strong association between having used a LDS syringe in the past 1 month and exposure to social marketing pilot activities, specifically LDSS promotion messaging in the two pilot provinces where LDS syringes were previously unknown and unused by PWID—Hanoi and Thai Nguyen. Respondents in these two Northern provinces that reported exposure to PSI’s LDS syringe promotion messages were found to have 21 times the likelihood of having used a LDS syringe in the past 1 month, compared with those that were not exposed to these messages.

In addition to changes in the use of LDS syringes, we found significant and positive differences in awareness of LDS syringes in Hanoi and HCMC, particularly in Hanoi, where awareness of LDS syringes more than doubled during the first year of pilot activities. The absence of baseline data in Thai Nguyen province disallows us from drawing conclusions about increases over the life of the intervention there. This is said given the fact that a relatively high number of PWID in Thai Nguyen report being aware of LDS syringes and using the product, its proximity to Hanoi, and the near non-existent presence of LDS syringes in pharmacies and other outlets in the north of Vietnam found in the initial market assessment; it is likely that the pilot program contributed to substantial increases in LDS syringe awareness and use in Thai Nguyen province as well.

These findings lead us to support the conclusion drawn by Vickerman et al. that availability in the market of LDS syringes may not, in itself, generate use at a level that can substantially impact on HIV transmission and prevalence [3]. Significant investment in efforts to improve convenient access including late-night availability, day-time accessibility (i.e., non-stigmatized ability to purchase single N/S), affordability, and informed demand for LDS syringes are also needed. Furthermore, we conclude that retailers are an essential component of LDS syringe social marketing interventions, particularly in contexts like Vietnam where almost all syringes are purchased at commercial outlets of some kind. Understanding when and where syringes are obtained—as well as insights regarding motivations and drivers of traders who operate these outlets—is the key to the future success of interventions that target a change in the types of syringes used by PWID. Changing the attitudes and practices of pharmacy operators and exploring additional, non-pharmacy outlets where LDS syringes can be accessed after pharmacies close are also important features of the pilot program. Affordability of LDS syringes and their availability late at night were both found to be positively and independently associated with LDS syringe use.

We also found that awareness of non-health benefits of LDS syringes had a stronger association with LDS syringe use than awareness of the product’s potential to reduce risk of HIV transmission. This suggests that product features that are not directly related to health are an important factor in shaping choices about types of syringes used and are important in future social marketing campaigns and behavior change interventions.

A further conclusion that we would draw from this work is that the scale of demand creation activities in relation to the size of the PWID population is key. The three pilot sites vary in the estimated size of their PWID populations: 26,821 in Hanoi, 25,573 in HCMC, and 6118 in Thai Nguyen [14]. We found relatively low coverage of demand creation activities relative to the total PWID population in Hanoi (16.7 %). In HCMC, coverage was 10.1 %. Conversely, in Thai Nguyen, where the estimated population of PWID is four times smaller than in Hanoi and HCMC, we saw high levels of coverage, with over 50 % reporting exposure to demand creation activities promoting LDS syringes. This high level of coverage was accompanied by relatively high levels of use of LDS syringes compared with Hanoi.

The sales of LDS syringes in HCMC reflect the fact that established distribution channels and strong commercial sales existed there prior to the pilot program. Because of this, PSI activities in HCMC gave greater focus to pharmacy behavior change and sales to non-traditional outlets to improve LDS syringe access late at night, when pharmacies are closed.

The magnitude of the sales increases observed across the pilot sites, together with the changes in awareness, access, and use of LDS syringes, lead us to conclude that future efforts to change the types of syringes used by PWID should take account of both supply and the demand-related barriers that may limit use. Raising awareness of product benefits beyond a reduction in risk of HIV and HCV transmission is key; we found that, in HCMC, where LDS syringe use is the norm, relatively few respondents were aware of the potential reduced risk of HIV transmission that may result from use. Almost all were aware of the wider product benefits—reduced drug loss, reduced scarring, and reduced pain. Promotion of the product benefits that do not relate directly to a reduced risk of HIV and HCV transmission should be considered a key part of future social marketing campaigns supporting LDS syringe use.

## Conclusions

We echo the conclusion drawn by other authors that there is a need for cohort studies that track HIV and HCV incidence in contexts where users are switching from high to low dead space syringes or where it would be possible to observe evidence of lower infection rates that could be attributed to consistent use of LDS syringes.

LDS syringes are a technology that has the potential to reduce HIV and HCV transmission among a key risk population in the HIV epidemic in large parts of Asia. The results of this 1-year pilot program demonstrate the potential of social marketing interventions that use marketing techniques to change behavior in a way that supports the introduction of this technology, to reduce HIV and HCV transmission and to save lives. As noted in a forthcoming UNAIDs report, investment is needed to ensure access and use of HIV prevention products if Vietnam is to achieve its objectives of being free of HIV by 2030 [15].

### Study limitations

The study uses repeated cross-sectional surveys from three provinces where the LDS syringe social marketing pilot program was implemented. No control/comparison population was possible due to limited time and resources. We acknowledge that this limits our ability to distinguish trends in LDS syringe awareness and use related to the pilot program from secular trends related to the same indicators.

All measures of LDS syringe awareness and use are based on self-reported data. As such, there is a possibility that some respondents may not present an accurate recall of events.

Finally, we acknowledge that, while statistical adjustments give RDS samples the characteristics of a probability sample, we cannot rule out the possibility that samples may be biased by respondent recruitment of participants.

## Abbreviations

CSOs: civil social organizations
HCMC: Ho Chi Minh City
HCV: hepatitis C virus
HDSS: high dead space syringes
HIV: human immunodeficiency virus
LDSS: low dead space syringes
NSP: needle and syringe programs
N/S: needle and syringe
PSI: Population Services International
PWID: people who inject drugs

## References

[1] Bobashev GV, Zule WA (2010). Modeling the effect of high dead-space syringes on the human immunodeficiency virus (HIV) epidemic among injecting drug users. Addiction.

[2] Walsh N, Verster A, Rodolph M, Akl EA (2014). WHO guidance on the prevention of viral hepatitis B and C among people who inject drugs. Int J Drug Policy.

[3] Vickerman P, Martin NK, Hickman M (2013). Could low dead space syringes really reduce HIV transmission to low levels?. Int J Drug Policy.

[4] Zule WA, Desmond DP, Neff JA (2002). Syringe type and drug injector risk for HIV infection: a case study in Texas. Soc Sci Med.

[5] Zule WA, Bobashev G (2009). High dead-space syringes and the risk of HIV and HCV infection among injecting drug users. Drug Alcohol Depend.

[6] Zule WA (2012). Low dead-space syringes for preventing HIV among people who inject drugs: promise and barriers. Curr Opin HIV AIDS.

[7] Gyarmathy VA, Neaigus A, Li N, Ujhelyi E, Caplinskiene I, Caplinskas S (2010). Liquid drugs and high dead space syringes may keep HIV and HCV prevalence high–a comparison of Hungary and Lithuania. Eur Addict Res.

[8] Ibragimov U. & Latypov A. Needle and syringe types used by people who inject drugs in Eastern Europe and Central Asia: key findings from a rapid assessment. Eurasian Harm Reduction Network, 2012.

[9] Zule WA, Cross HE, Stover J, Pretorius C (2013). Are major reductions in new HIV infections possible with people who inject drugs? The case for low dead-space syringes in highly affected countries. Int J Drug Policy.

[10] PSI/Vietnam: LDSS rapid assessment, 2012.

[11] PSI/Vietnam: behavioral survey among PWID, 2012.

[12] Grund JP, Friedman SR, Stern LS, Jose B, Neaigus A, Curtis R (1996). Syringe-mediated drug sharing among injecting drug users: patterns, social context and implications for transmission of blood-borne pathogens. Soc Sci Med.

[13] Heckathorn D (2002). Respondent driven sampling II: deriving valid population estimates from chain-referral samples of hidden populations. Soc Probl.

[14] Ministry of Health: Vietnam HIV/AIDS estimates and projections 2007–2012.

[15] Ministry of Health & UNAIDS: Optimizing Vietnam’s HIV response: an investment case 2014.

